# Supplementary material for: Eleven key measures for monitoring general practice clinical activity during COVID-19: A retrospective cohort study using 48 million adults’ primary care records in England through OpenSAFELY
Source: eLife. 2023 Jul 27;12:e84673. doi: 10.7554/eLife.84673 (PMC10374277; doi:10.7554/eLife.84673)
Supplement: Supplementary file 1. [file elife-84673-supp1.pdf]

Supplementary Table 1. Counts of the five most commonly used codes within each measure codelist between January 2019 and April 2021.

Measure	Code	SNOMED-CT Top Level Hierarchy	Description	Proportion of codes, TPP (%)	Proportion of codes, EMIS (%)	Total Proportion of codes (%)
Blood pressure monitoring	72313002	Observable entity	Systolic arterial pressure	95.6	96.7	96.2
	413606001	Observable entity	Average home systolic blood pressure	2.9	2.3	2.5
	407554009	Observable entity	Sitting systolic blood pressure	0.5	0.3	0.4
	400974009	Observable entity	Standing systolic blood pressure	0.5	0.3	0.3
	314445006	Observable entity	Average night interval systolic blood pressure	0.1	-	0.1
	314449000	Observable entity	Average 24 hour systolic blood pressure	-	0.2	-
Cardiovascular Disease 10 Year Risk Assessment	718087004	Observable entity	QRISK2 cardiovascular disease 10 year risk score	98.9	95.1	96.5
	763244005	Observable entity	QRISK cardiovascular disease 10 year risk calculator score	0.8	3.7	2.6
	1085871000000105	Observable entity	QRISK3 cardiovascular disease 10 year risk calculator score	0.3	0.9	0.6
	809311000000105	Procedure	Cardiovascular disease risk assessment by third party	<0.1	0.3	0.2
	752451000000100	Observable entity	Framingham coronary heart disease 10 year risk score	-	0.1	0.1

	752451000000100	Observable entity	Joint British Societies cardiovascular risk score	<0.1	-	-
Cholesterol Testing	1005671000000105	Observable entity	Serum cholesterol level	99.1	96.9	97.9
	1017161000000104	Observable entity	Plasma total cholesterol level	0.8	3.1	2.0
	850981000000101	Observable entity	Cholesterol level	0.2	<0.1	0.1
	853681000000104	Observable entity	Total cholesterol level	-	<0.1	<0.1
	365794002	Finding	Finding of serum cholesterol level	-	<0.1	<0.1
	395153009	Procedure	Pre-treatment serum cholesterol level	<0.1	-	-
Liver Function Testing - Alanine aminotransferase (ALT)	1018251000000107	Observable entity	Serum alanine aminotransferase level	96.4	95.6	96.0
	1013211000000103	Observable entity	Plasma alanine aminotransferase level	3.5	4.2	3.8
	250637003	Procedure	Alanine aminotransferase - blood measurement	0.1	0.3	0.2
	34608000	Procedure	Alanine aminotransferase measurement	0.1	-	<0.1
	201321000000108	Procedure	Serum alanine aminotransferase level	-	<0.1	<0.1
	390961000	Procedure	Plasma alanine aminotransferase level	-	<0.1	-
Thyroid Testing (TSH)	1022791000000101	Observable entity	Serum thyroid stimulating hormone level	97.6	97.1	97.3
	1022801000000102	Observable entity	Plasma thyroid stimulating hormone level	2.3	2.7	2.5
	1027151000000105	Observable entity	Thyroid stimulating hormone level	0.1	0.2	0.2
	61167004	Procedure	Thyroid stimulating hormone measurement	-	<0.1	<0.1

	313440008	Procedure	Measurement of serum thyroid stimulating hormone	-	<0.1	<0.1
Full Blood Count - Red Blood Cell (RBC) Testing	1022451000000103	Observable entity	Red blood cell count	100.0	>99.9	>99.9
	365625004	Finding	Finding of red blood cell count	-	<0.1	<0.1
	14089001	Procedure	Red blood cell count	-	<0.1	<0.1
Glycated Haemoglobin A1c Level (HbA1c)	999791000000106	Observable entity	Haemoglobin A1c level - IFCC standardised	98.9	94.5	96.5
	1003671000000109	Observable entity	Haemoglobin A1c level	1.1	5.5	3.5
	43396009	Procedure	Hemoglobin A1c measurement	0.1	<0.1	<0.1
	365845005	Finding	Hemoglobin A1C - diabetic control finding	<0.1	<0.1	<0.1
	313835008	Procedure	Haemoglobin A1c measurement aligned to the Diabetes Control and Complications Trial	-	<0.1	<0.1
Renal Function Assessment - Sodium Testing	1000661000000107	Observable entity	Serum sodium level	97.8	96.8	97.2
	1017381000000106	Observable entity	Plasma sodium level	2.2	3.2	2.8
Asthma Reviews	394700004	Procedure	Asthma annual review	63.6	60.8	62.1
	394720003	Procedure	Asthma medication review	17.4	8.4	12.5
	401182001	Procedure	Asthma monitoring by nurse	9.2	8.6	8.9
	270442000	Finding	Asthma monitoring check done	-	11.0	5.9
	401183006	Procedure	Asthma monitoring by doctor	-	5.1	2.7

	394701000	Procedure	Asthma follow-up	4.8	-	-
	390877003	Procedure	Step up change in asthma management plan	2.6	-	-
Chronic Obstructive Pulmonary Disease (COPD) Reviews	394703002	Procedure	Chronic obstructive pulmonary disease annual review	93.9	98.7	96.5
	760621000000103	Procedure	Chronic obstructive pulmonary disease 6 monthly review	3.5	0.9	2.1
	760601000000107	Procedure	Chronic obstructive pulmonary disease 3 monthly review	2.7	0.4	1.4
Medication Review	314530002	Situation	Medication review done	69.3	8.0	34.7
	182836005	Procedure	Review of medication	3.0	42.7	25.4
	88551000000109	Procedure	Medication review with patient	2.9	19.6	12.3
	93311000000106	Procedure	Medication review of medical notes	-	12.5	7.1
	394720003	Procedure	Asthma medication review	2.5	2.0	2.3
	719329004	Situation	Medication review done by pharmacist	3.1	-	-

“-”: code outside the top five codes
